# Supplementary figures and images for: Investigation on Adsorption of Polar Molecules in Vegetable Insulating Oil by Functional Fossil Graphene
Source: Materials (Basel). 2023 Apr 28;16(9):3434. doi: 10.3390/ma16093434 (PMC10180333; doi:10.3390/ma16093434)

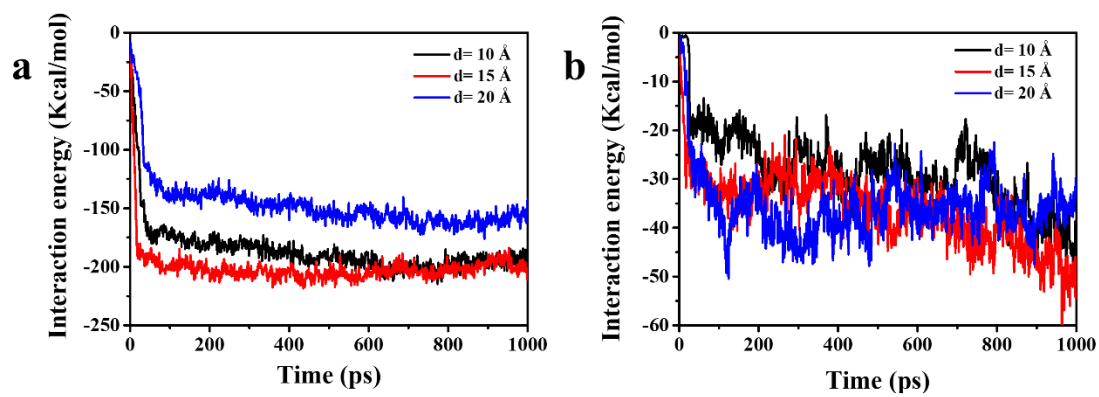

Figure S1 Interaction energy of graphene systems with different pore sizes

Supplement: Supplementary file 1 [file materials-16-03434-s001.zip › materials-2319404-supplementary.pdf]
